# Quality assessment and dissemination of varicocele health information on short-video platforms: A comparative study of TikTok and Bilibili

**DOI:** 10.1097/MD.0000000000048359

**Published:** 2026-04-10

**Authors:** Pan Ding, Qinghua Luo, Leihua Cao

**Affiliations:** a Department of Urology, Nanchang People’s Hospital, Nanchang, Jiangxi, China.

**Keywords:** Bilibili, digital health, health communication, information quality, short-video platforms, TikTok, varicocele

## Abstract

TikTok and Bilibili have gradually become important sources for the public to obtain health information. This study aims to evaluate the content, quality, and reliability of varicocele-related videos. We conducted a search on both platforms using the keyword “varicocele” and collected the top 150 videos based on the default rankings, along with video duration, engagement metrics, uploader identity, and video content. Videos were assessed using the global quality score (GQS) and modified DISCERN (mDISCERN) scales. Mann–Whitney *U* tests and Kruskal–Wallis tests were used to compare differences between groups. Spearman correlation analysis was employed to evaluate the relationships between video features, engagement, and quality. A total of 255 videos were included. The content of the videos was primarily focused on treatment (71.37%), with limited coverage of prevention (32.16%). The median GQS score was 3.00 (interquartile range (IQR): 3.00–4.00), and the median mDISCERN score was 2.00 (IQR: 2.00–2.00). TikTok demonstrated a higher GQS score than Bilibili (*P* < .05). Compared to individual users and non-specialists, videos uploaded by specialists scored higher on both GQS and mDISCERN (*P* < .05). No significant correlation was found between engagement metrics and either GQS or mDISCERN (*P* > .05). The quality and reliability of varicocele-related videos are suboptimal, with insufficient coverage of prevention content. Videos uploaded by specialists demonstrated higher quality and reliability. Engagement metrics were not correlated with video quality or reliability. Future platforms should strengthen content monitoring and review processes and provide support for professional creators to enhance the credibility and educational value of digital health communication.

## 1. Introduction

Varicocele is among the most common vascular disorders of the male reproductive system, characterized by abnormal dilation and tortuosity of the pampiniform plexus, typically resulting from venous valvular incompetence or impaired venous return.^[1]^ Varicocele occurs most frequently on the left side and affects roughly 15% of men in the general population. While some patients conceive without treatment, prevalence increases substantially among infertile cohorts 35 to 44% in primary infertility and 45 to 81% in secondary infertility.^[2,3]^ Despite being asymptomatic in a subset of patients, varicocele is closely associated with testicular atrophy, impaired semen parameters, and reduced androgen production, and is recognized as a leading cause of reversible male infertility.^[4–6]^ Given its high prevalence and the potential long-term consequences for reproductive health, demand from the public and patients for accessible, reliable information on diagnosis and management continues to grow.

With the rise of digital media, short-video platforms (e.g., TikTok and Bilibili) – characterized by rapid dissemination, strong visual appeal, and highly fragmented content have become major channels through which the public acquires medical knowledge and health information.^[7]^ This mode of access can enhance disease awareness, shorten care-seeking delays, and promote early recognition and guideline-concordant treatment. However, in the absence of unified medical review, some content driven by “traffic-oriented” incentives may be exaggerated, incomplete, or inaccurate, resulting in uneven information quality and, at times, misleading the public.^[8,9]^

Previous studies on video content related to pulmonary nodules, irritable bowel syndrome, and pancreatic cancer have found that these videos often have high engagement, but their quality and reliability are generally moderate.^[10–12]^ These findings indicate that dissemination popularity does not necessarily correspond to informational accuracy or clinical value. Nevertheless, disease-specific evidence within the field of urology and andrology remains limited. In particular, despite the high prevalence and clinical importance of varicocele, systematic evaluations focusing on the quality, content coverage, and dissemination characteristics of varicocele-related short videos are scarce, and cross-platform comparative analyses are rarely reported.

Given the clinical significance of varicocele, including its association with male infertility, testicular atrophy, and impaired semen quality, which can affect reproductive health and quality of life, it is crucial to analyze the quality and reliability of varicocele-related videos on Bilibili and TikTok.This study used TikTok and Bilibili as sample platforms to analyze Chinese short video content related to varicocele, assessing content quality, medical coverage, and dissemination characteristics, while exploring the associations between uploader attributes and content performance. The aim of this study is to identify strengths and weaknesses in current digital health communication and to provide preliminary, evidence-based references for platform governance, professional content production, and patient education strategies, thereby improving the quality and reliability of varicocele-related health information.

## 2. Methods

### 2.1. Study design, data sources, and search strategy

This study conducted searches on Bilibili and TikTok on September 10th using the keyword “varicocele.” To minimize the influence of recommendation algorithms, new accounts were created on both platforms. Browsing histories and caches were cleared prior to the search. The top 150 videos were collected according to the platforms’ default comprehensive rankings.

### 2.2. Inclusion and exclusion criteria

Inclusion criteria were: video content related to varicocele, language in Chinese or English, short video format that could be played independently, and adequate audio and visual quality. Exclusion criteria were: irrelevant content, and duplicate uploads.

### 2.3. Data extraction

For each video, we extracted platform, duration in seconds, numbers of likes, favorites, comments, and shares, uploader type classified as specialists, non-specialists, or individual user, and content category. Content categories were assigned according to 6 core medical dimensions that included epidemiology, etiology, clinical presentation, diagnosis, treatment, and prevention.

### 2.4. Video quality and reliability assessment

Video quality was evaluated using the global quality scale (GQS), scored from 1 to 5 (Table 1), which captures structural clarity, informational completeness, and overall viewing value.^[13]^ Reliability was assessed with the modified DISCERN (mDISCERN) instrument, comprising 5 binary items with a total score of 0 to 5 (Table 2), reflecting the credibility of information sources and the provision of references.^[14]^ Two medically trained researchers rated all videos independently after standardized training. In case of disagreement, a third reviewer will adjudicate.

**Table 1:** The global quality score (GQS) quality criteria.

Item features	Points
Poor quality; poor flow of the videos; most information missing; not at all useful for patients	1
Generally poor quality; some information listed, but many important topics missing; of very limited use to patients	2
Moderate quality; suboptimal flow; some important adequately discussed, but other information poorly discussed; somewhat useful for patients	3
Good quality and generally good flow; most of the relevant information listed, but some topics not covered; useful for patients	4
Excellent quality and flow; very useful for patients	5

GQS = global quality scale.

**Table 2:** The modified DISCERN (mDISCERN) quality criteria.

Reliability score
1. Is the video clear, concise, and understandable?
2. Are valid sources cited?
3. Is the content presented balanced and unbiased?
4. Are additional sources of content listed for patient reference?
5. Are areas of uncertainty mentioned?

mDISCERN = modified DISCERN.

### 2.5. Video content scoring

To quantify the depth of medical coverage, we developed a 6 dimension content scoring framework encompassing epidemiology, etiology, clinical presentation, diagnosis, treatment, and prevention. Each dimension was scored from 0 to 2 based on whether the topic was mentioned, the accuracy of the information, and the completeness of the description, yielding a total score of 0 to 12.^[15]^ Scoring procedures mirrored those for quality assessment, with 2 independent raters applying uniform criteria. In cases of notable disagreement, a third reviewer adjudicated.

### 2.6. Statistical analysis

Descriptive statistics were used to summarize the data. Continuous variables were reported as medians and interquartile ranges, and categorical variables were presented as frequencies and percentages. The Mann–Whitney *U* test was used for comparisons between platforms. Comparisons between uploader types were made using the Kruskal–Wallis test. Spearman rank correlation analysis was performed to examine associations among engagement metrics, quality scores, and content scores. All tests were two-sided, and a *P*-value <.05 was considered statistically significant. All statistical analyses and plotting were performed using R version 4.3.3.

## 3. Results

### 3.1. Video characteristics

**Figure 1.**
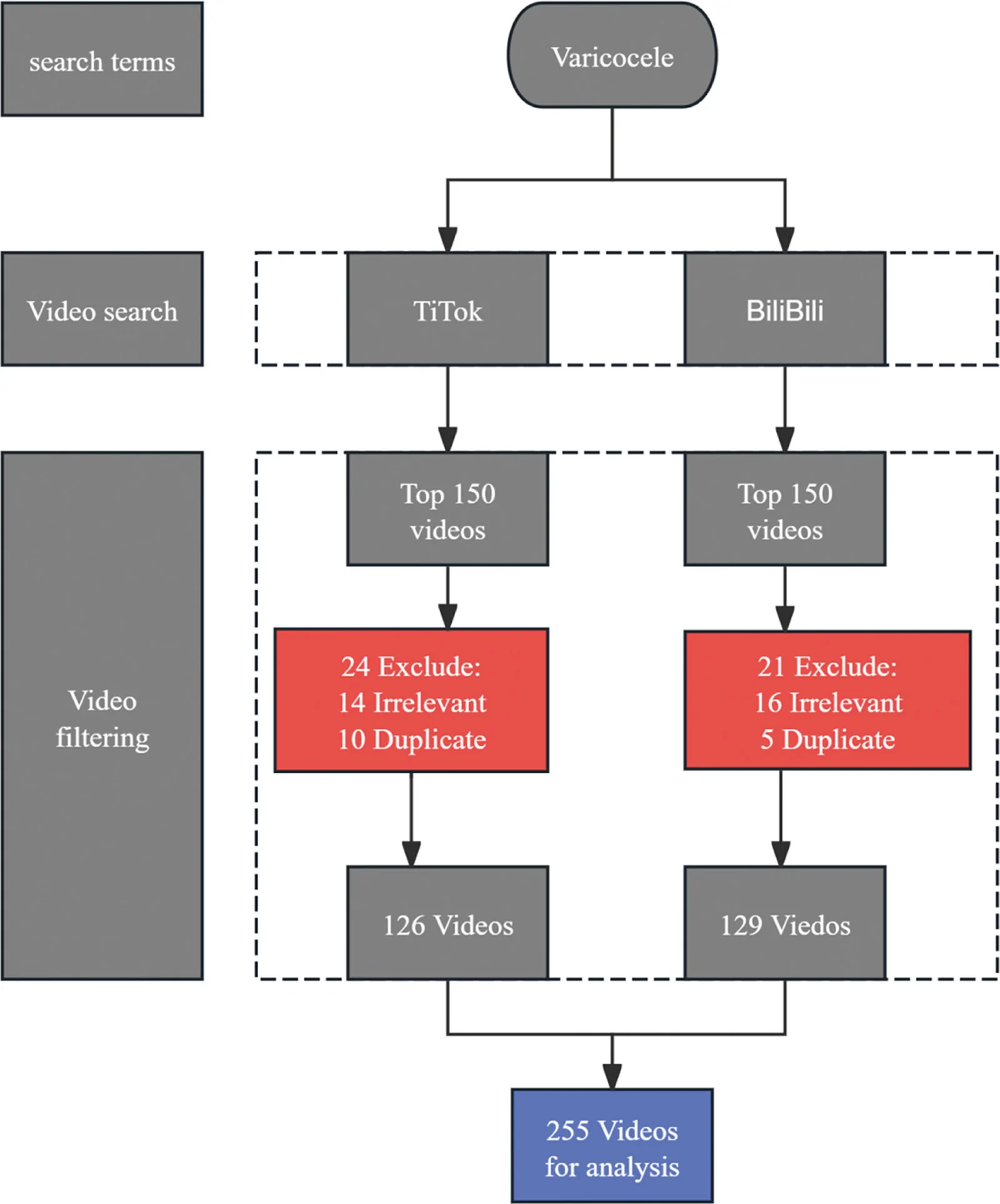
Flowchart of the study. Notation: Short videos on varicocele were identified on TikTok and Bilibili using the keyword “varicocele.” The top 150 videos per platform were screened. On TikTok, 24 were excluded (14 irrelevant, 10 duplicates), leaving 126 eligible videos; on Bilibili, 21 were excluded (16 irrelevant, 5 duplicates), leaving 129 eligible videos. In total, 255 videos were included for analysis.

A total of 255 varicocele-related short videos were included, comprising 126 from TikTok and 129 from Bilibili. Each platform initially yielded 150 videos; after screening for relevance and duplicates, 24 TikTok and 21 Bilibili videos were excluded (Fig. 1).

Overall, the median video duration was 58 seconds (interquartile range (IQR): 41–98). Engagement metrics showed a median of 215 likes (IQR: 9.5–1227.0), 95 favorites (IQR: 6.0–361.5), 18 comments (IQR: 2.0–157.5), and 46 shares (IQR: 1.0–491.5). Regarding content categories, treatment-oriented videos predominated (71.37%), whereas prevention was least represented (32.16%). Quality assessments indicated moderate overall quality but limited reliability, with a median GQS of 3.00 (IQR: 3.00–4.00) and a median mDISCERN of 2.00 (IQR: 2.00–2.00), as summarized in Table 3.

**Table 3:** General information, quality, and reliability scores of varicocele-related videos on TikTok and BiliBili.

Variables	Total (n = 255)
General information	
Video length(s), M (Q_1_, Q_3_)	58.00 (41.00, 98.00)
Likes, M (Q_1_, Q_3_)	215.00 (9.50, 1227.00)
Collections, M (Q_1_, Q_3_)	95.00 (6.00, 361.50)
Comments, M (Q_1_, Q_3_)	18.00 (2.00, 157.50)
Shares, M (Q_1_, Q_3_)	46.00 (1.00, 491.50)
Video content (n)%	
Epidemiology	114 (44.71%)
Etiology	133 (52.16%)
Clinical manifestation	170 (66.67%)
Diagnosis	136 (53.33%)
Treatment	182 (71.37%)
Prevention	82 (32.16%)
Video quality	
GQS score, M (Q_1_, Q_3_)	3.00 (3.00, 4.00)
mDISCERN, M (Q_1_, Q_3_)	2.00 (2.00, 2.00)
Uploader n(%)	
Individual user	35 (13.72%)
Non-specialists	60 (23.53%)
Specialists	160 (62.75%)

GQS = global quality score, M = median, mDISCERN = modified DISCERN, Q_1_ = 1st quartile, Q_3_ = 3rd quartile.

### 3.2. Comparative analysis of engagement, content, and quality across platforms

TikTok outperformed Bilibili across all engagement metrics, including likes (989.00 vs 12.00), favorites (266.50 vs 7.00), comments (89.00 vs 3.00), and shares (407.00 vs 2.00) with all differences statistically significant (*P* < .05).

Video duration was slightly shorter on TikTok than on Bilibili (56.50 s vs 60.00 s, *P* < .05). Median mDISCERN scores were similar between platforms, although their distributions differed markedly. GQS was higher on TikTok than on Bilibili (*P* < .05). Regarding content categories, TikTok included a greater proportion of videos on epidemiology (59.52% vs 30.23%) and prevention (67.46% vs 27.90%), whereas Bilibili emphasized clinical presentation and treatment. Detailed comparisons are provided in Table 4.

**Table 4:** Comparison of characteristics between different short-video platforms.

Variables	Bilibili (n = 129)	Tiktok (n = 126)	*P*
Video length(s), M (Q_1_, Q_3_)	60.00 (43.00, 167.00)	56.50 (39.00, 84.00)	<.05
Likes, M (Q_1_, Q_3_)	12.00 (2.00, 120.00)	989.00 (327.50, 2382.00)	<.05
Collections, M (Q_1_, Q_3_)	7.00 (1.00, 103.00)	266.50 (88.75, 679.25)	<.05
Comments, M (Q_1_, Q_3_)	3.00 (0.00, 40.00)	89.00 (13.75, 237.00)	<.05
Shares, M (Q_1_, Q_3_)	2.00 (0.00, 27.00)	407.00 (88.50, 1020.00)	<.05
mDISCERN, M (Q_1_, Q_3_)	2.00 (2.00, 5.00)	2.00 (2.00, 2.75)	<.05
GQS score, M (Q_1_, Q_3_)	3.00 (2.00, 3.00)	3.00 (3.00, 4.00)	<.05
Video content (n)%, M (Q_1_, Q_3_)	3.00 (2.00, 5.00)	4.00 (2.00, 6.00)	<.05
Epidemiology	39 (30.23%)	75 (59.52%)	–
Etiology	55 (42.64%)	78 (61.9%)	–
Clinical manifestation	86 (74.42%)	84 (66.67%)	–
Diagnosis	74 (57.36%)	70 (55.56%)	–
Treatment	98 (75.97%)	84 (66.67%)	–
Prevention	36 (27.9%)	85 (67.46%)	–
Uploader (n)%			
Individual user	27 (20.93%)	8 (6.35%)	<.05
Non-specialists	35 (27.13%)	25 (19.84%)	
Specialists	67 (51.94%)	93 (73.81%)	

GQS = global quality score, M = median, mDISCERN = modified DISCERN, Q_1_ = 1st quartile, Q_3_ = 3rd quartile.

### 3.3. Uploader type distribution

**Figure 2.**
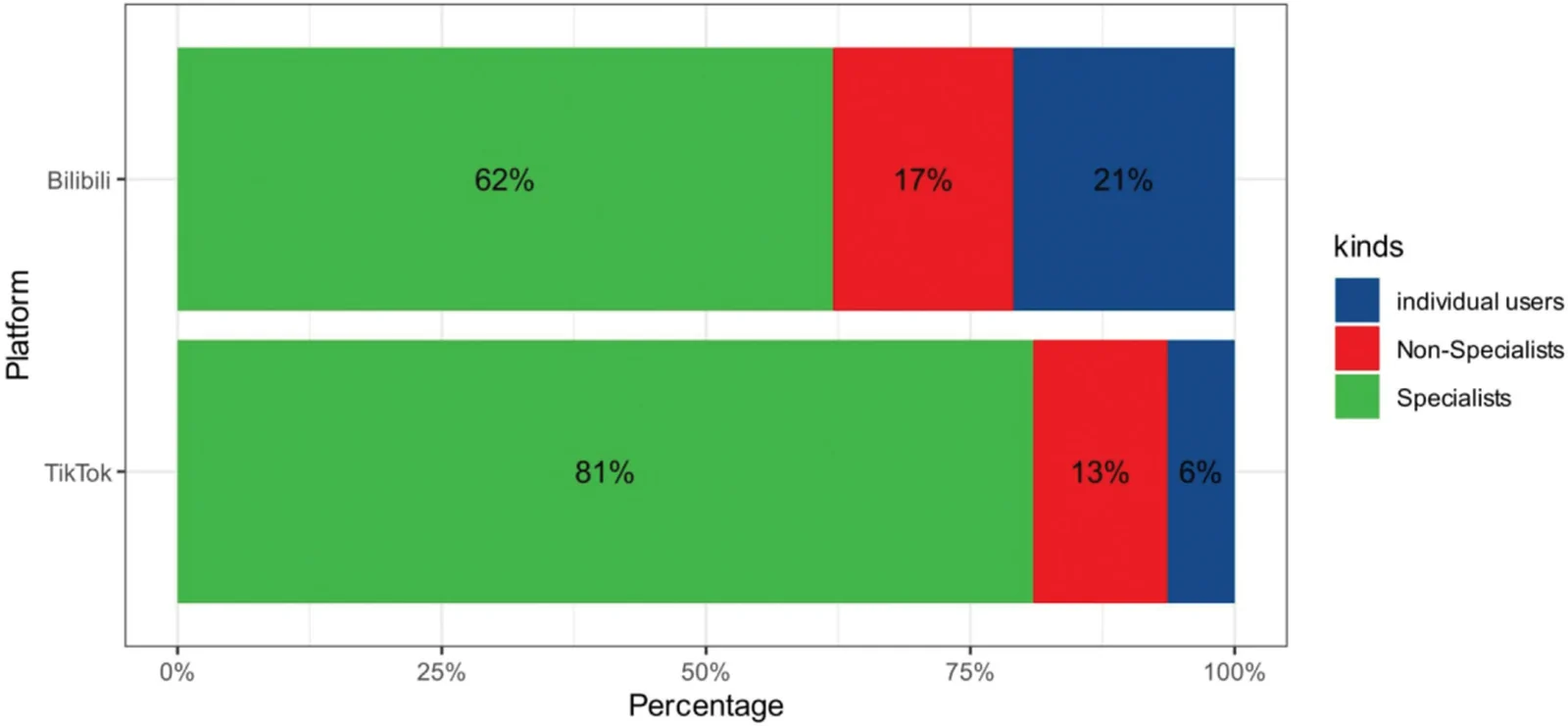
Distribution of videos by uploader type on TikTok and Bilibili platforms.

As shown in Figure 2, a larger proportion of TikTok videos were uploaded by specialists (81%), whereas Bilibili featured higher shares of non-specialists (27%) and individual users (21%), indicating marked differences in creator composition between the 2 platforms.

### 3.4. Distribution of video content themes

**Figure 3.**
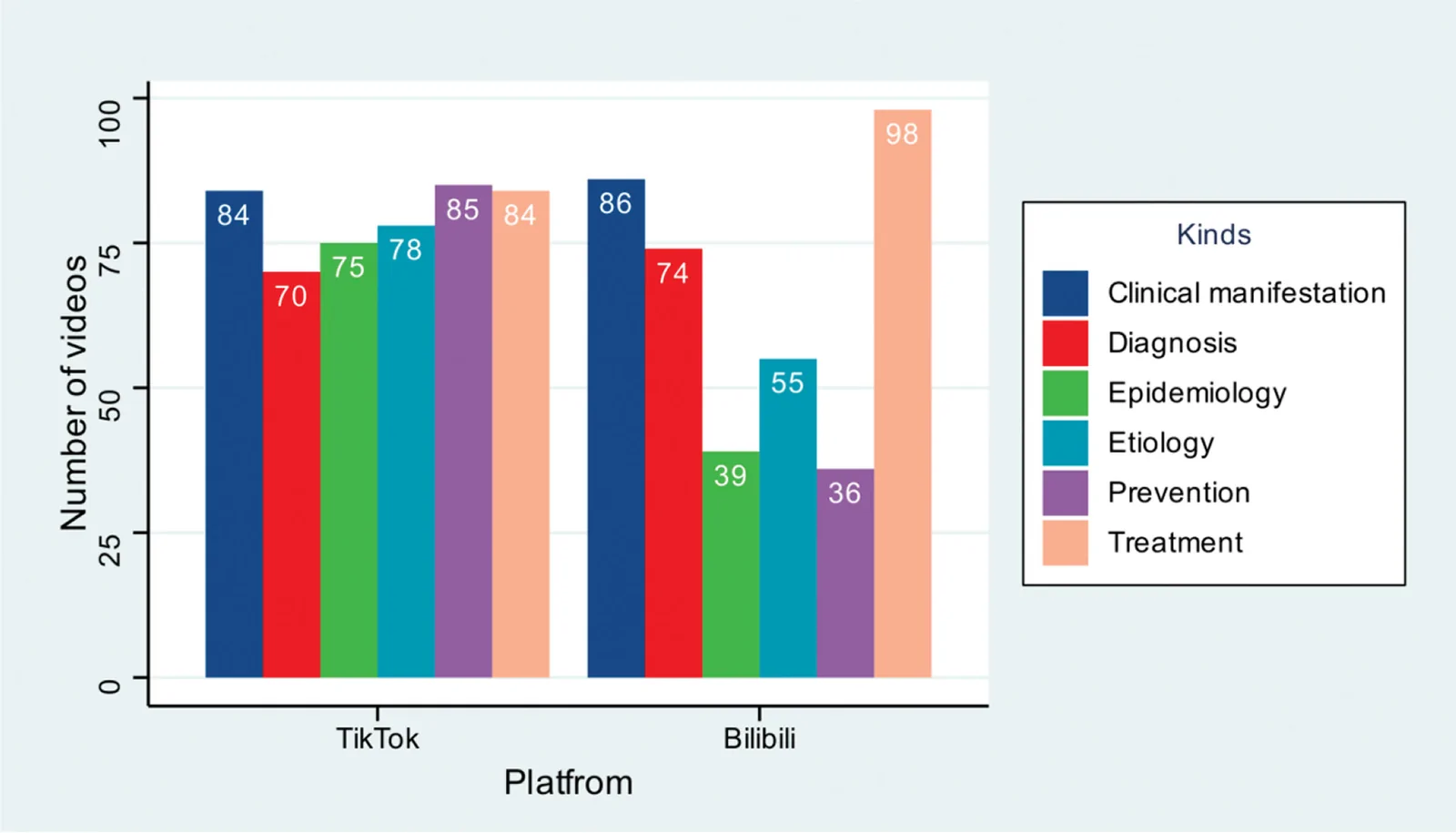
Distribution of video content categories on TikTok and Bilibili platforms.

Table 3 summarized the distribution of varicocele-related videos: treatment was the most common theme (71.37%), followed by clinical manifestation (66.67%) and diagnosis (53.33%), whereas prevention was least represented (32.16%). Figure 3 depicted platform-specific distributions. On TikTok, content was more evenly distributed across the 6 medical themes (e.g., treatment 84 videos and epidemiology 75 videos), whereas Bilibili concentrated more heavily on treatment (98 videos).

### 3.5. Quality comparison by uploader

**Figure 4.**
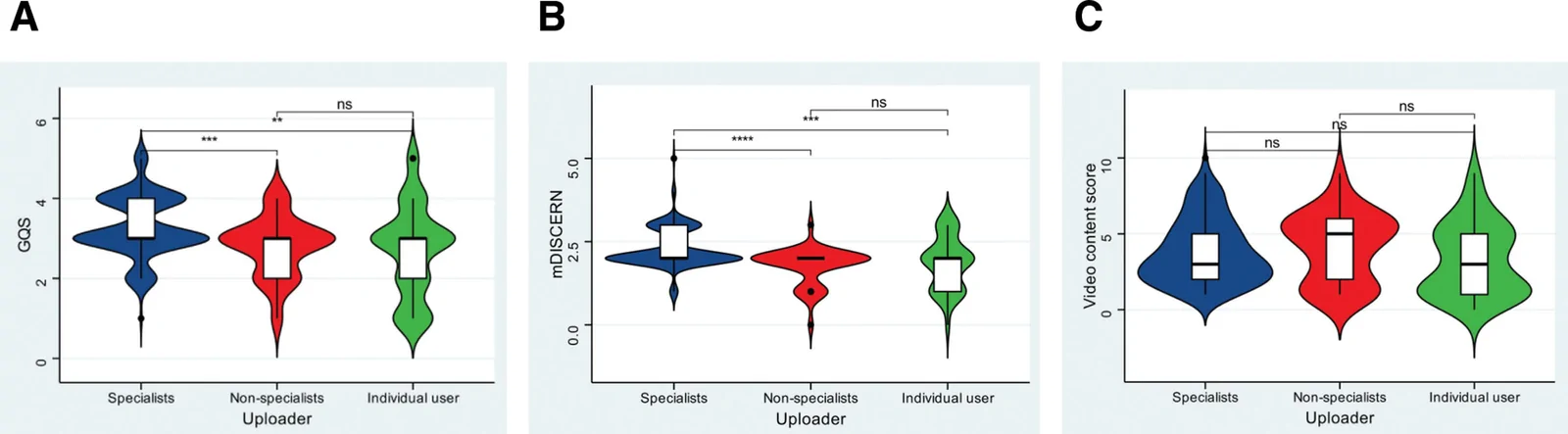
Uploader-type differences in quality (GQS), reliability (mDISCERN), and content scores. Notation: Violin plots with embedded boxplots compare (A) global quality scale (GQS, 1–5), (B) modified DISCERN (mDISCERN, 0–5), and (C) video content score (0–12) across uploader types (specialists, non-specialists, individual users). Boxes depict medians and interquartile ranges; whiskers represent 1.5 × IQR. Pairwise differences were tested with the Mann–Whitney *U* test; ** indicates *P* < .01 and ns indicates not significant. Higher scores reflect better video quality, source reliability, and content completeness.

Figure 4(A–C) compare differences across uploader types in GQS, mDISCERN, and content completeness scores. Compared with individual users and non-specialists, specialists achieved higher scores on both GQS and mDISCERN (both *P* < .05). Detailed parameters are presented in Table 5.

**Table 5:** Comparison of mDISCERN, video content, and GQS scores across different uploader categories.

Variables	Individual user (n = 35)	Non-specialists (n = 38)	Specialists (n = 182)	*P*
mDISCERN, M (Q_1_, Q_3_)	2.00 (1.00, 2.00)	2.00 (2.00, 2.00)	2.00 (2.00, 3.00)	<.05
Video content score, M (Q_1_, Q_3_)	3.00 (1.00, 5.00)	5.00 (3.00, 6.00)	3.00 (2.00, 5.00)	<.05
GQS score, M (Q_1_, Q_3_)	3.00 (2.00, 3.00)	3.00 (2.00, 3.00)	3.00 (3.00, 4.00)	<.05
Video length, M (Q_1_, Q_3_)	121.00 (54.00, 270.00)	79.50 (54.75, 162.75)	53.00 (39.00, 80.00)	<.005
Likes, M (Q_1_, Q_3_)	111.00 (14.50, 491.50)	117.50 (11.25, 1262.00)	295.00 (7.00, 1323.00)	>.05
Collections, M (Q_1_, Q_3_)	38.00 (5.50, 430.50)	86.00 (10.00, 362.75)	95.50 (6.00, 353.75)	>.05
Comments, M (Q_1_, Q_3_)	10.00 (0.00, 228.50)	13.00 (2.25, 156.00)	28.00 (2.00, 153.75)	>.05
Shares, M (Q_1_, Q_3_)	27.00 (1.00, 223.50)	30.50 (3.00, 681.25)	65.00 (1.00, 543.50)	>.05

GQS = global quality score, M = median, mDISCERN = modified DISCERN, Q_1_ = 1st quartile, Q_3_ = 3rd quartile.

### 3.6. Comparison of video quality scores between platforms

**Figure 5.**
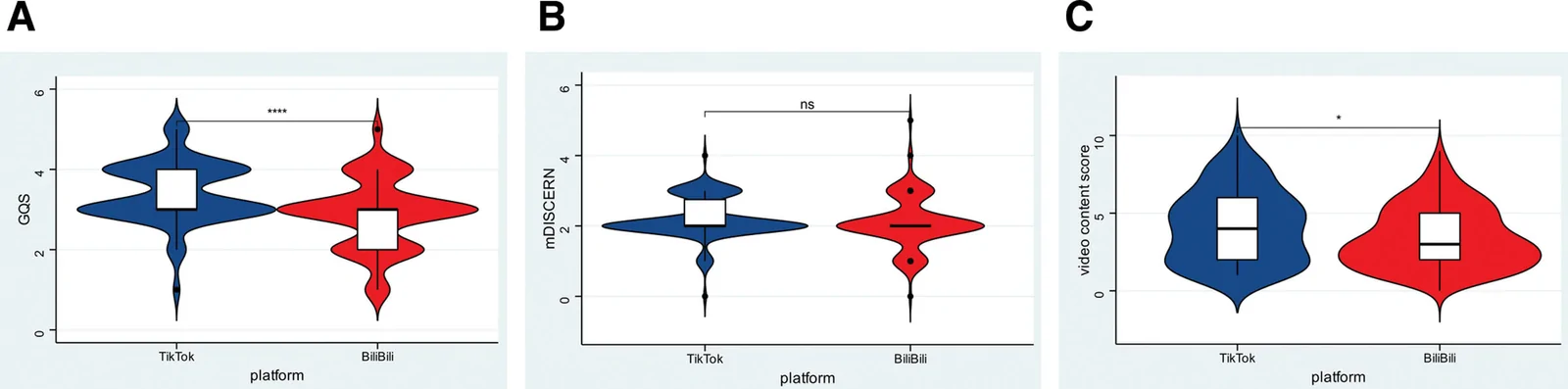
Platform-level differences in GQS, mDISCERN, and video content score. Notation: Violin plots with embedded boxplots compare TikTok (n = 126) and Bilibili (n = 129) for (A) global quality scale (GQS, 1–5), (B) modified DISCERN (mDISCERN, 0–5), and (C) video content score (0–12). TikTok showed higher GQS (**** *P* < .0001) and a higher content score (* *P* < .05), whereas no significant difference was observed for mDISCERN (ns). Boxes indicate medians and interquartile ranges; whiskers denote 1.5 × IQR. Pairwise comparisons used the Mann–Whitney *U* test; higher values indicate better quality/reliability/completeness.

TikTok demonstrated a higher GQS score than Bilibili (*P* < .05; Fig. 5A), while no between-platform difference was observed for mDISCERN (*P* > .05; Fig. 5B). The video content-completeness score was also higher on TikTok (*P* < .05; Fig. 5C). Detailed parameters for GQS, mDISCERN, and content completeness across platforms are summarized in Table 4.

### 3.7. Correlation between video characteristics and quality scores

**Figure 6.**
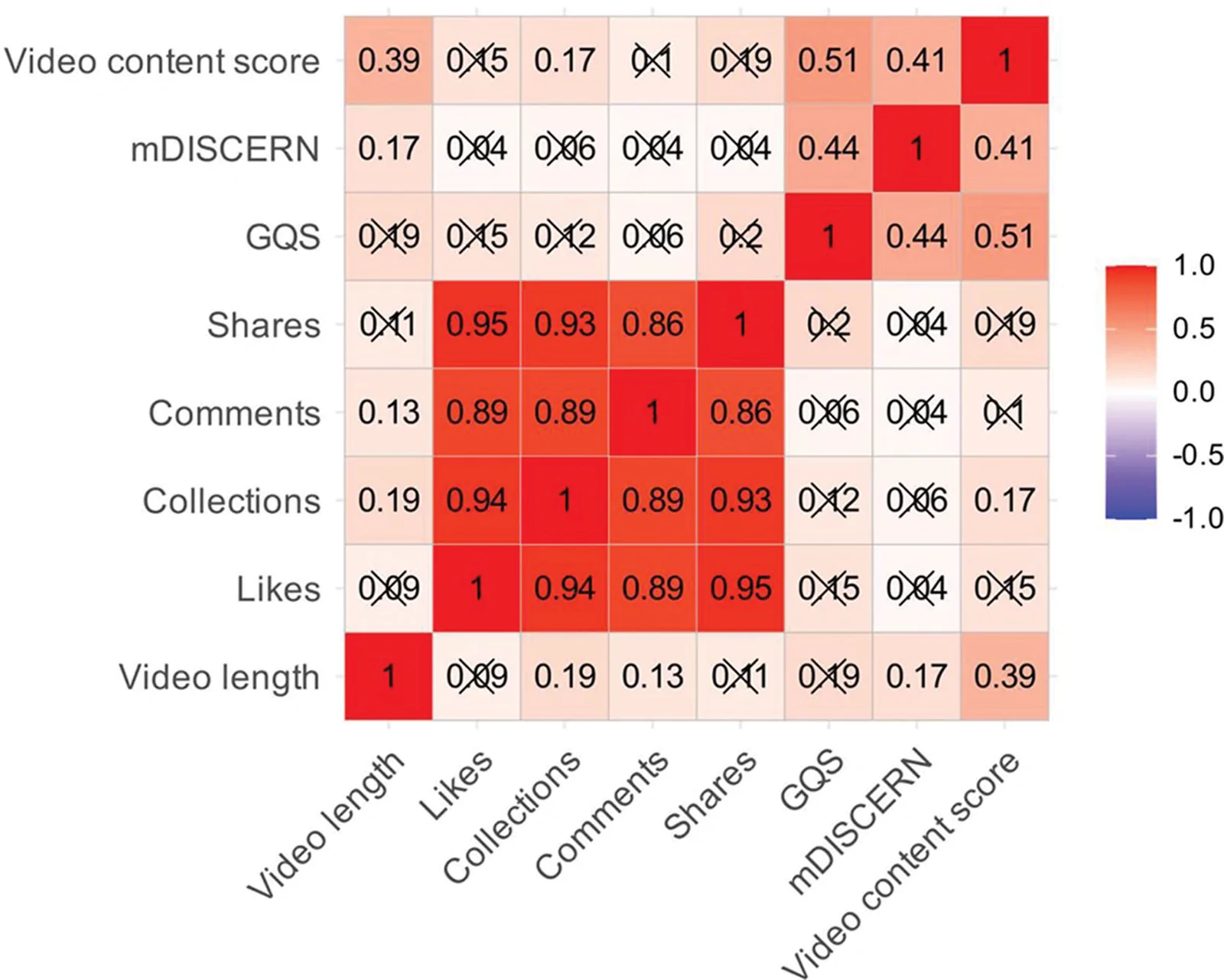
Correlation matrix of video engagement metrics and quality scores on TikTok and Bilibili. Notation: Matrix shows pairwise Spearman ρ between video length, likes, collections (favorites), comments, shares, global quality scale (GQS), mDISCERN, and the video content score. Cell color encodes correlation magnitude from −1 (blue) to +1 (red); overlaid numbers are ρ values; white crosses indicate non-significant associations (two-tailed, *P* > .05). Engagement metrics were highly inter-correlated, GQS correlated moderately with mDISCERN, the content score correlated moderately with GQS/mDISCERN but only weakly with engagement, and video length showed weak correlations overall.

Spearman correlation analyses showed strong inter-correlations among engagement metrics – likes with collections (*R* = 0.94), likes with comments (*R* = 0.89), likes with shares (*R* = 0.95), comments with shares (*R* = 0.86), comments with collections (*R* = 0.89), and shares with collections (*R* = 0.93). GQS and mDISCERN were moderately and positively correlated (*R* = 0.44). The video content score correlated moderately with GQS (*R* = 0.51) and mDISCERN (*R* = 0.41), but only weakly with engagement indicators (likes, comments, collections/favorites, shares: *R* = 0.09–0.19) and with video length (*R* = 0.39). Notably, engagement metrics did not show a significant association with either GQS or mDISCERN (Fig. 6).

## 4. Discussion

Drawing on 2 major short video platforms, TikTok and Bilibili, this study analyzed 255 varicocele related videos across engagement metrics, content domains, informational quality, and reliability. These findings indicate that short video based health communication exerts substantial reach but still requires improvement in scientific rigor and systematic content organization. The results provide practical guidance for optimizing digital health education strategies by highlighting the need to strengthen evidence based content development, broaden topic coverage beyond treatment oriented messaging, and promote creator training and verification mechanisms that can enhance both credibility and educational value.

Overall, TikTok substantially outperformed Bilibili on all engagement indicators, including likes, comments, favorites, and shares Notably, the median number of likes on TikTok was 989.00, far exceeding Bilibili’s 12.00. Prior studies suggest that such disparities can be attributed to TikTok’s recommendation logic, which prioritizes entertainment value and short, high frequency interaction cycles, thereby amplifying audience reach and user participation. This pattern is consistent with earlier analyses of TikTok’s dissemination effects, which indicate that short videos are readily elevated to high exposure positions by platform algorithms, resulting in high engagement and high traffic dynamics.^[10,11,16]^ Bilibili videos are significantly longer than those on TikTok (60.00 seconds vs 56.50 seconds, *P* < .05), reflecting divergent content strategies and audience expectations. Bilibili tends to support more structured and explanatory knowledge delivery, permits longer durations, and emphasizes depth of learning. TikTok prioritizes short form dissemination efficiency and affective triggers, focusing on rapid attention capture and immediate interaction. These patterns are consistent with prior research in which Bilibili content resembles a microlecture format that facilitates knowledge construction, whereas TikTok more often adopts an information dispatch style aimed at short term impact and broad diffusion.^[7,17]^ Therefore, although TikTok is more active on engagement metrics, Bilibili may be better suited to fostering deeper understanding and knowledge retention. Future health communication should tailor content length and structural design to platform characteristics, balancing dissemination efficiency with information quality.

From a content classification perspective, treatment related videos constituted the largest share at 71.37 percent, whereas coverage of epidemiology at 44.71 percent and prevention at 32.16 percent was clearly inadequate. This distribution suggests an emphasis on immediate management while overlooking early detection, risk factor education, and behavioral interventions, which may contribute to delayed presentation and, in some cases, serious complications. It may further lead the public to adopt clinical recommendations without sufficient understanding of etiology and population trends, thereby weakening prevention awareness and self management. Coverage of prevention was significantly higher on TikTok than on Bilibili at 67.46 percent versus 27.90 percent, likely reflecting platform incentives that favor concise and actionable messaging.^[15]^ A narrow content structure has become a common limitation of short video based health education, and prior studies of diabetes and hypertension related short videos have reached similar conclusions.^[18,19]^ Future efforts should establish standardized content frameworks to encourage creators to balance the proportions of treatment and prevention information, thereby improving the comprehensiveness of public health education.

From the overall quality assessment, both platforms show a median mDISCERN score of 2.0 and a median GQS score of 3.0, indicating persistent deficiencies in source citation, evidentiary completeness, and clarity of presentation. Consistent with prior evaluations of short video content on childhood pneumonia and mammography, short video platforms frequently lack explicit source attribution and structured content design, with visual appeal often prioritized over informational completeness.^[20,21]^ This study further shows that the proportion of Specialists is significantly higher on TikTok than on Bilibili at 74 percent versus 52 percent, and videos on TikTok achieve superior scores on both mDISCERN and GQS, with all differences reaching statistical significance. These findings suggest that professional participation contributes to greater informational reliability and overall visual quality. Nevertheless, even on TikTok, overall quality remains at a moderate level and reliability is suboptimal, indicating that uploader background alone is insufficient to ensure standards are met. A plausible explanation is that TikTok places greater emphasis on entertainment and message simplification, which may over compress professional content during dissemination and thereby limit the depth of exposition.^[22–24]^ Platforms should build on professional verification by implementing structured authoring guidance and content review mechanisms to enhance the systematization and credibility of health information.

In this study, videos posted by specialists achieved significantly higher mDISCERN and GQS scores than those posted by non-specialists and individual users, and their content score distributions were more concentrated, indicating more complete information structure and more standardized expression. These findings align with the conclusions of Li and colleagues in multi-platform evaluations of health video quality, which identified medical background as a key factor determining informational credibility.^[25]^ Nevertheless, even content uploaded by professionals did not reach high quality thresholds at the median, indicating that content review and authoring guidance still require strengthening. Going forward, platforms should raise entry requirements for non specialists, implement expert verification mechanisms, and incentivize healthcare institutions to proactively provide high quality content, thereby enhancing the credibility of the overall health information ecosystem.

Spearman correlation analyses showed strong associations among engagement indicators, whereas the correlations between engagement and video quality, and between video length and engagement, were weak. In addition, the association between video length and quality scores was not significant, indicating that content depth is not necessarily proportional to duration. This aligns with prior research suggesting that short video formats can convey high quality information within limited time, provided that structure and content planning are well designed.^[26]^ Furthermore, engagement indicators were not significantly correlated with GQS, mDISCERN, or content completeness. This disconnection suggests that highly engaging videos may not necessarily reflect accurate or high-quality health information, potentially facilitating the rapid dissemination of misinformation. Platforms should avoid duration bias when designing recommendation algorithms and instead incorporate quality-related factors such as structural clarity and source citation into their ranking metrics, thereby improving algorithmic logic and enhancing the visibility of high-quality content.

Through a multidimensional analysis of dissemination patterns, content distribution, and quality levels of varicocele related short videos, this study shows that short video based health communication offers clear advantages in information accessibility but remains limited in content structure, informational depth, and source reliability. Although user behavior differs markedly between platforms, quality scores are similar, indicating that professional content still requires platform level mechanisms and strategic support. Uploader background exerts a significant influence on content quality, underscoring the need for certification systems and guidance that favor professional contributors. These findings provide important implications for optimizing the health communication functions of short video platforms and for building a trustworthy ecosystem for digital health education.

This study has several limitations. First, the sample was limited to TikTok and Bilibili, excluding overseas platforms such as YouTube and Instagram, which may limit the cross-cultural generalizability. Moreover, differences in platform governance mechanisms, content moderation policies, and user demographics across regions may further constrain the external applicability of the findings. Second, the sample size was relatively small, and the representativeness of the sample may be limited. Although a standardized search strategy was applied and top-ranked videos were systematically collected to reduce selection bias, the included sample may still not fully capture the entire spectrum of varicocele-related content available on these platforms. Third, although validated scales were used, subjectivity was inevitably introduced. Despite the implementation of standardized rating criteria, independent assessments by 2 medically trained reviewers, and adjudication by a third reviewer in cases of disagreement, a certain degree of subjective judgment in evaluating video quality, reliability, and content completeness could not be completely eliminated. Lastly, this study is a cross-sectional study and cannot reflect the dynamic changes in the content, quality, and reliability of varicocele-related information. Given the rapid turnover of short-video content and frequent updates driven by platform recommendation algorithms, longitudinal monitoring would be necessary to better characterize temporal trends and evolving patterns of health information dissemination.

Future research should build upon these findings by incorporating longitudinal designs to capture temporal changes in the quality, reliability, and thematic focus of varicocele-related short-video content, as well as by expanding analysesto additional international platforms to improve cross-cultural generalizability. Methodologically, the integration of automated or artificial intelligence–assisted assessment tools may complement manual evaluations and reduce subjectivity. Importantly, greater involvement of urologists and andrologists in short-video content creation, guided by evidence-based standards, transparent source citation, and structured educational frameworks, together with collaboration among professional societies, academic institutions, and platform administrators, may help promote higher-quality and more reliable health communication.

## 5. Conclusion

This study evaluated the content quality and dissemination characteristics of videos related to varicocele on TikTok and Bilibili. Despite high user engagement, overall informational quality and reliability remained suboptimal, with content heavily concentrated on treatment and insufficient coverage of prevention. Compared with individual users, videos posted by specialists demonstrated higher quality and reliability. Platforms are encouraged to strengthen guidance and review mechanisms for scientific content and to optimize health information dissemination strategies. Future efforts should encourage greater participation of specialists in varicocele-focused public education to improve the overall quality of related videos.

## Acknowledgments

The authors would like to express their sincere gratitude to the video uploaders on TikTok and Bilibili for their contributions to public health by sharing health-related information.

## Author contributions

**Conceptualization:** Pan Ding, Qinghua Luo.

**Data curation:** Pan Ding, Qinghua Luo, Leihua Cao.

**Formal analysis:** Leihua Cao.

**Funding acquisition:** Leihua Cao.

**Investigation:** Qinghua Luo, Leihua Cao.

**Methodology:** Pan Ding.

**Project administration:** Leihua Cao.

**Resources:** Pan Ding.

**Software:** Pan Ding.

**Supervision:** Qinghua Luo, Leihua Cao.

**Validation:** Pan Ding, Qinghua Luo.

**Visualization:** Pan Ding.

**Writing – original draft:** Pan Ding.

**Writing – review & editing:** Pan Ding, Qinghua Luo, Leihua Cao.
